# PPV distribution of sidewalls induced by underground cavern blasting excavation

**DOI:** 10.1038/s41598-021-86055-y

**Published:** 2021-03-23

**Authors:** Yi Luo, Xiaoqing Wei, Junhong Huang, Guang Zhang, Xing Bian, Xinping Li

**Affiliations:** 1 Hubei Key Laboratory of Road-Bridge and Structure Engineering, Wuhan University of Technology, Wuhan, China; 2 School of Civil Engineering and Architecture, Wuhan University of Technology, Wuhan, China; 3 School of Safety Science and Emergency Management, Wuhan University of Technology, Wuhan, China

**Keywords:** Civil engineering, Power stations, Petrology

## Abstract

The peak particle velocity (PPV) is an important indicator for predicting blasting excavation disturbances. However, the PPV distribution in the deep underground space is significantly different from that on the outdoor ground. Therefore, it is difficult to predict the underground PPV by Sadovsky’s vibration formula. The PPV sidewall distribution characteristics were studied during site blasting in an underground cavern in the Taohuazui mine in China, and a similar numerical model was used to verify the site test data. We derived a PPV prediction formula for the underground cavern sidewall surrounding rock using a mechanical analysis model of a simply supported plate and beam in combination with dimensional analysis. The model considered derived boundary constraints, comparison with site measured data, the value predicted by Sadovsky’s vibration formula, and numerical simulation results. The results showed that the PPV distribution on the middle 1/3 section of the underground cavern sidewall showed a “platform” or “bulge” different from the curve from Sadovsky’s vibration formula. The PPV amplification coefficient in this section was distributed in a drum shape. The PPV prediction formula for the middle section of the sidewall derived in this paper was highly consistent with the data measured on-site and the numerical simulation results. The mechanical analysis model with a simply supported plate and beam included an underground cavern sidewall length–height ratio of 5 and effectively supplemented the PPV prediction formula for the middle section of the traditional underground cavern sidewall.

## Introduction

To date, drilling-blasting is still the main excavation method for constructing tunnels, mines, underground hydropower stations, and other underground spaces. Researchers continue to study blasting safety during construction^1–4^. With rapid developments in the scale and buried depth of underground excavation^5^, the construction of a large number of underground caverns has been commenced successively, and the associated difficulties in blasting excavation construction and vibration prediction control of upright high sidewalls have been increasing^6–9^. During blasting excavation, energy propagates along the sidewall in the form of vibration waves, inducing damage to the surrounding rock and lining and leading to sidewall instability, closed function failure, and other problems. This greatly increases the construction safety risk and operation maintenance costs. Engineering practices have shown that there is a good correlation between the PPV and structural failure^10^. A large number of studies on the attenuation law of PPV and its fitting formulas have emerged, such as empirical prediction formulas represented by Sadovsky’s cube-root formulas^11^ and USBM square-root formulas^12^. Therefore, the PPV of underground cavern sidewalls should be strictly and accurately tested and predicted^13^.

The theoretical and empirical formulas derived by Sadovsky from spherical charge initiation conditions in an infinite free field (i.e., Sadovsky’s vibration formula) have significant limitations when used to predict the PPV distribution of blasting vibrations in underground jointed rock masses^14–17^. Many researchers have studied the slope elevation effect under blasting vibration^18–22^ and proposed modified Sadovsky’s formulas. These modifications use a power function or an approximate curve to predict the PPV monotonicity attenuation with the distance from the blasting centre or survey point elevation^23^. Besides, many scholars have developed a series of soft computing methods based on modern computer technology for PPV prediction model, such as artificial neural network^24^, gray relational analysis^25^ and genetic programming^26^. Compared with the traditional empirical prediction formulas, through the soft science method, generally more input parameters can be considered, and the predicted value and measured value can have a higher degree of fitting through intelligent algorithm based learning and training of the model^27^. Table 1 shows several recently-investigations with their performances in predicting PPV using soft computing techniques. Other scholars used a large amount of PPV measured data to train different algorithm models with better blasting vibration PPV prediction^28–32^.

**Table 1:** Some studies of PPV prediction using soft computing methods^33–39^.

Technique	Input parameter	No. of dataset	R^2^
SVM	DI, C	80	R^2^ = 0.96
ICA-ANN	BS, ST, C, DI, Vp, E	95	R^2^ = 0.98
ANN	C, PF	232	R^2^_ANN_ = 0.92
ANFIS			R^2^_ANFIS_ = 0.98
CART	C, DI	86	R^2^ = 0.95
FS-ICA	W, D	50	R^2^ = 0.94
GMDH	SC, W, D	96	R^2^_GMDH_ = 0.91
GS-GMDH			R^2^_GS-GMDH_ = 0.94
ANFIS-GOA	PF, ST, RD, S, B	80	R^2^_ANFIS-GOA_ = 0.97
ANFIS-CA			R^2^_ANFIS-CA_ = 0.95

*SVM* support vector machine, *ICA* imperialist competitive algorithm, *ANN* artificial neural network, *ANFIS* adaptive neuro-fuzzy inference system, *CART* classification and regression tree, *FS* fuzzy system, *GMDH* Group Method of Data Handling, *GS* generalized structure, *GOA* grasshopper optimization algorithm, *CA* cultural algorithm, *DI* distance from the blasting face, *C* charge per delay, *BS* burden to spacing, *ST* stemming, *Vp* P-wave velocity, *E* Young modulus, *PF *powder factor, *W* weight charge per delay, *D* distance from the blasting point, *SC* specific charge, *RD* rock density, *S* spacing, *B* burden, *R*^*2*^ coefficient of determination.

The boundary constraints of underground upright sidewall planes or cylindrical sidewalls are significantly different from those of the infinite space considered by Sadovsky’s vibration formula or the semi-infinite space on open ground. Many studies have proven that the vibration attenuation of underground structure blasting is different from that of surface blasting^40–42^. Therefore, describing underground sidewall PPV simply by the amplification effect has limitations due to the lack of a reasonable mechanical model for dynamic analysis and a formula suitable for underground engineering practices. As a result, the industry requires a PPV prediction formula suitable for underground high sidewalls. Some scholars believed that, different from blasting vibration propagation in continuous ground media, blasting vibration attenuation in underground projects is affected by special spatial geometry and constraints, and attempted to consider the high sidewall as a simply supported beam for vibration modal and mode analysis, but no in-depth study or discussion was carried out^43,44^.

Based on the blasting excavation project of the underground space in Taohuazui mine of China, the mechanical calculation model of PPV prediction and amplification effect distribution characteristics of underground engineering blasting is established through the combination of in-situ test, theoretical analysis and numerical simulation. Combined with dimensional analysis method, the PPV prediction formula of surrounding rocks induced by underground engineering blasting excavation is obtained and the application scope of the simplified end constraint method is determined.

## PPV distribution sidewalls induced by underground blasting excavation

### Design of blasting test on the sidewall of an underground cavern in the Taohuazui mine

**Figure 1:**
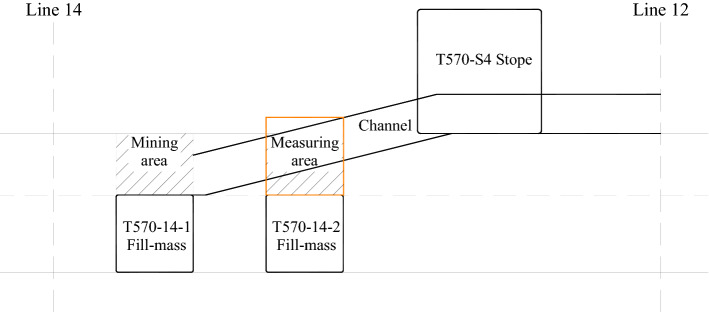
Section of horizontal line 12–14 slope at − 570 m.

The underground cavern in the Taohuazui mine located in the middle and lower reaches of the Yangtze River in Daye City, Hubei Province, China was chosen as the site for this test in consideration of its spatial structure and surrounding rock characteristics representative of underground projects. Geological conditions of surrounding rocks in the underground cavern: the orebody is simple in shape and lenticular as a whole. The ore is a skarn bearing copper–gold ore, with the main minerals of pyrite and chalcopyrite, followed by magnetite and siderite, and a small amount of bornite. Generally, the ore is compact and massive. The upper and lower walls of the ore block are skarn or skarn with quartz syenite diorite porphyritic. The surrounding rock of this cavern was highly intact and unsupported, which reflected the vibration performance of the original cavern sidewall surrounding rock. The overall dimensions of the underground cavern were 32 m × 10 m × 12 m (L × W × H). The section of the underground cavern tested is shown in Fig. 1.

**Figure 2:**
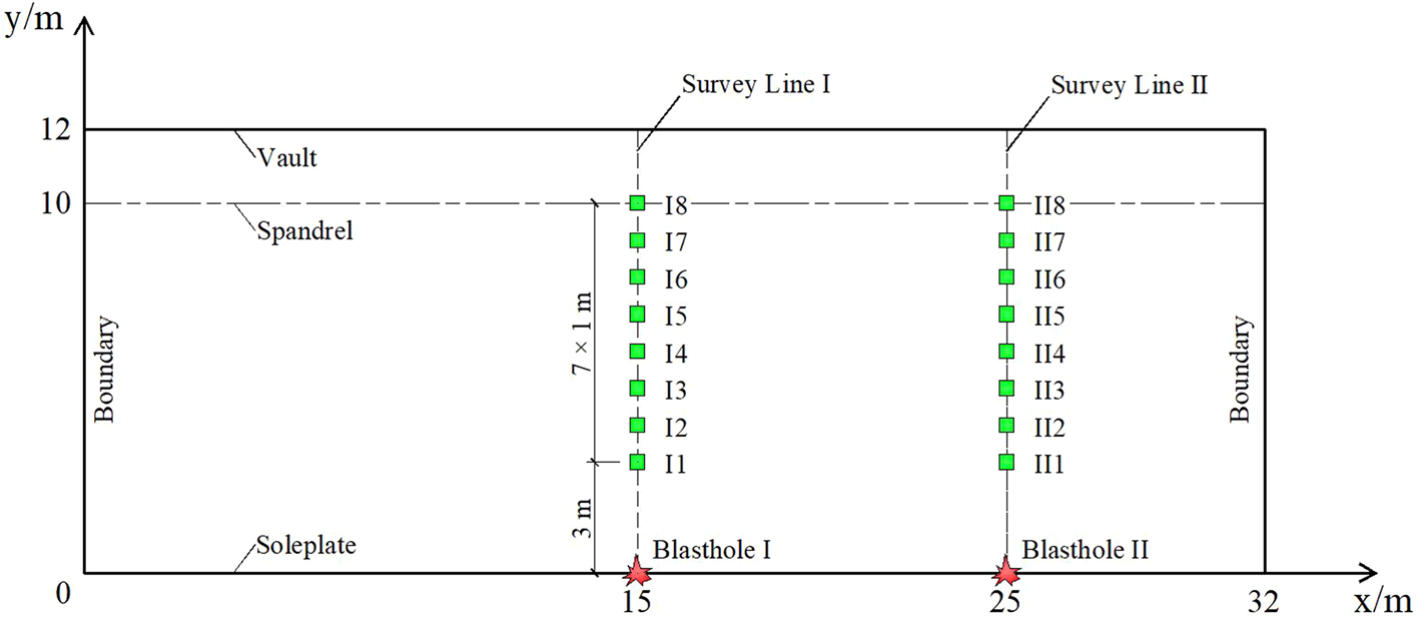
Sidewall blasting vibration survey points.

We conducted two blasts in this test and recorded the blasting vibration information on the two survey lines during each blast. We modelled the underground cavern sidewall surface as a simplified rectangle, and the intersection of the sidewall bottom plate and the left boundary was set to the origin. The bottom plate and the left boundary were defined as the X-axis and Y-axis, respectively, to establish a rectangular coordinate system as shown in Fig. 2. The blastholes were named Blasthole I and Blasthole II with coordinates in Fig. 2 of (15, 0) and (25, 0), respectively. The two blasts were performed in Blastholes I and II. The diameters of the blastholes and the cartridge were 38 mm and 32 mm, respectively. The depth of the blastholes was 2.0 m. The blastholes were charged with φ32 mm emulsion explosive. The maximum single shot dose was 4 kg.

**Figure 3:**
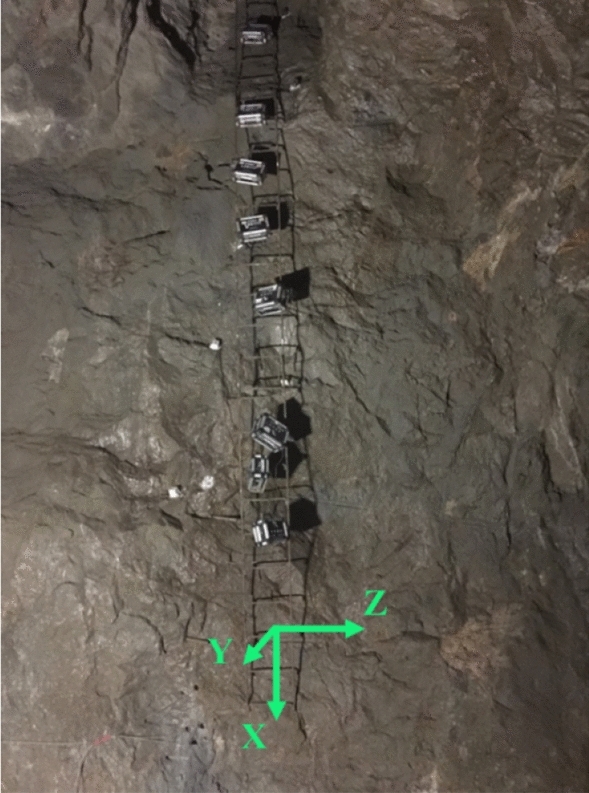
Installation drawing for site survey points.

The two survey lines arranged for the survey points in this test were named Survey Line I and Survey Line II as shown in Fig. 2. Eight survey points were arranged on each survey line along the sidewall surface from bottom to the top. The space between adjacent survey points was 1 m. The survey points at positions with a relative elevation difference of 3–10 m were named I1–I8 and II1–II8, respectively. PPV sensor installation at the site survey points is shown in Fig. 3.

### Analysis of measured PPV data

**Figure 4:**
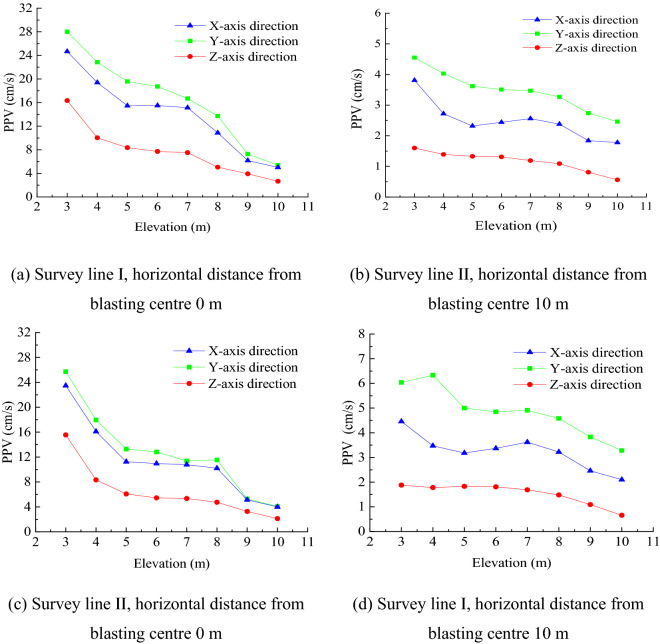
Variation of the measured sidewall PPV with elevation difference.

Blasthole I was initiated first to obtain the three-dimensional PPV of survey points I1-I8 is shown in Fig. 4a; then, the three-dimensional PPV of survey points II1-II8 was obtained and is shown in Fig. 4b. Next, Blasthole II was initiated to obtain the three-dimensional PPV of survey points II1-II8 (Fig. 4c) and I1-I8 (Fig. 4d).

According to Fig. 4, the PVV reached the maximum in the Y-axis direction perpendicular to the sidewall and the minimum in the horizontal Z-axis direction (i.e., $V_{Y} > V_{X} > V_{Z}$)_._ Therefore, the study was carried out on the PPV in the Y-axis direction perpendicular to the sidewall. Comparing the PPV of survey points on Survey Lines I and II under the initiation conditions of Blastholes I and II showed that the smaller the horizontal distance from the blasting centre, the higher the PPV. Although the PPV variation in the three directions did not completely monotonically decrease with increasing elevation difference, it showed an overall decreasing trend. The separate analysis of each set of data showed that the PPV in the upper section of the underground cavern sidewall varied with the elevation difference. The PPV in the middle section of the sidewall showed a “platform” or “bulge” generally distributed in the elevation range of 5–8 m. The data on the two blast test survey lines were compared with Sadovsky’s vibration formula (1) to verify whether the sidewall PPV had drum distribution.

1 $$V = k\left( {\frac{{\sqrt[3]{Q}}}{R}} \right)^{\beta }$$

First, the data measured on Survey Line I were used to determine the site coefficients in Sadovsky’s vibration formula: $k = 128.2$ and $\beta = 1.74$. The predicted PPV values on Survey Line I were obtained by substituting other known quantities into the formula. Finally, the measured PPV on Survey Line I was compared with the PPV predicted by Sadovsky’s vibration formula, and the ratio was defined as the amplification coefficient. Similarly, the PPV of the survey points on Survey Line II were fitted to obtain the Sadovsky’s vibration formula coefficients $k = 86.34$ and $\beta = 1.57$. The PPV on Survey Line II was predicted by Sadovsky’s vibration formula, and we obtained the amplification coefficient.

**Figure 5:**
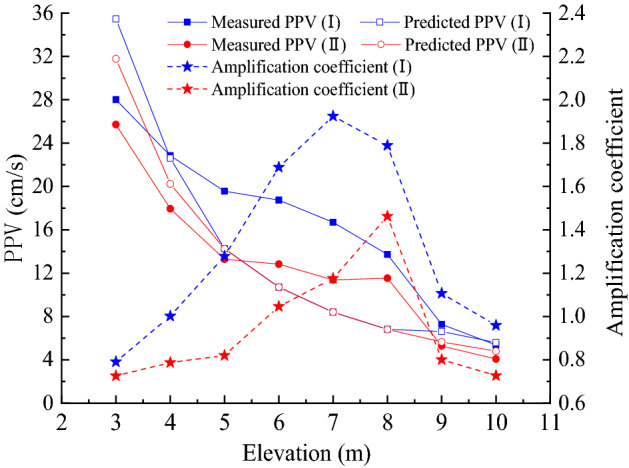
Elevation distribution curve for the sidewall PPV and amplification coefficient in the underground cavern in the Taohuazui mine.

In order to easily assess the amplification coefficient distribution of each survey point, the measured value in the Y-axis direction perpendicular to the sidewall, the value predicted by Sadovsky’s vibration formula, and the amplification coefficient were drawn in the same figure (Fig. 5). The PPV along the sidewall surface decayed with the elevation distribution as a whole. However, there was no obvious decrease in the PPV of the survey points within the relative elevation difference range of 5–8 m, but a “platform” or “bulge” indicated that the survey points near the middle elevation positions had an amplification effect. It can be seen by observing the peak value of the amplification coefficient curve that the maximum value of the amplification coefficient in the middle of the sidewall was close to 2.0. The amplification effects of Survey Lines I and II were different; the survey points on Survey Line I closer to the centre of the sidewall had a greater amplification effect, indicating that the degree of amplification was associated with the position. The closer to the centre of the sidewall, the greater the amplification effect.

Since the amount of site measured data was limited and the survey points had discrete distributions, further systematic analysis will be conducted in combination with ANSYS/LS-DYNA^45^, the numerical simulation software for dynamic finite elements.

### Numerical simulation of the on-site test

**Figure 6:**
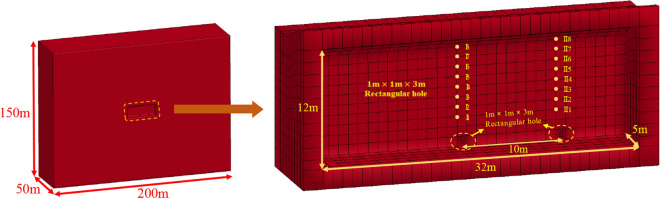
Diagram of the numerical model established.

**Figure 7:**
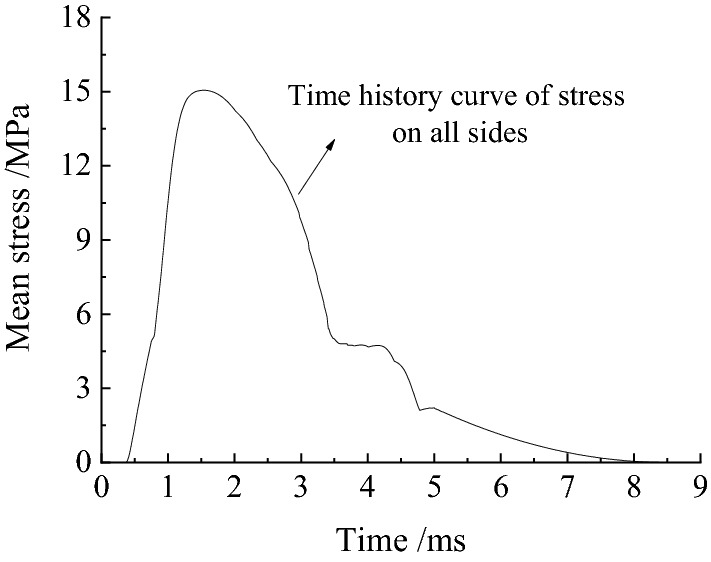
Mean stress on four sidewalls over time.

The model boundary dimensions are typically approximately 5–10 times the dimensions of the cavern when the analysis software for dynamic finite elements is used to analyse an underground cavern to prevent influence on the results. The underground cavern in the Taohuazui mine was used for this blasting excavation simulation. The boundary dimensions of the model were 200 m × 100 m × 150 m (L × W × H). The dimensions of this cavern were 32 m × 10 m × 12 m (L × W × H). The whole model included 535,028 units and 556,632 nodes, as shown in Fig. 6. Two rectangular holes (1 m × 1 m × 3 m) are reserved in the model to apply the equivalent explosion load. According to the charging parameters of the blasting holes in Fig. 2, the equivalent mean stress applied on the inner wall of the rectangular hole can be calculated. As shown in Fig. 7, the mean stress rose from 0 MPa, suddenly increased to the peak stress, and then decreased quickly. The maximum mean stress was 15.8 MPa at 1.56 ms. The mean stress decreased to 0 MPa at 8.5 ms. The mechanical parameters (as shown in Table 2) of the rock material were obtained from testing site rock samples.

**Table 2:** Rock material mechanical parameters.

Density ρ/(kg/m^3^)	Elasticity modulus E/(GPa)	Poisson’s ratio μ	Yield strength σ/MPa	Tangent modulus E_t_/GPa	Compressive strength R_c_/MPa	Firmness coefficient f
2700	53.3	0.21	100	8.0	120	5.80 ~ 12.55

**Figure 8:**
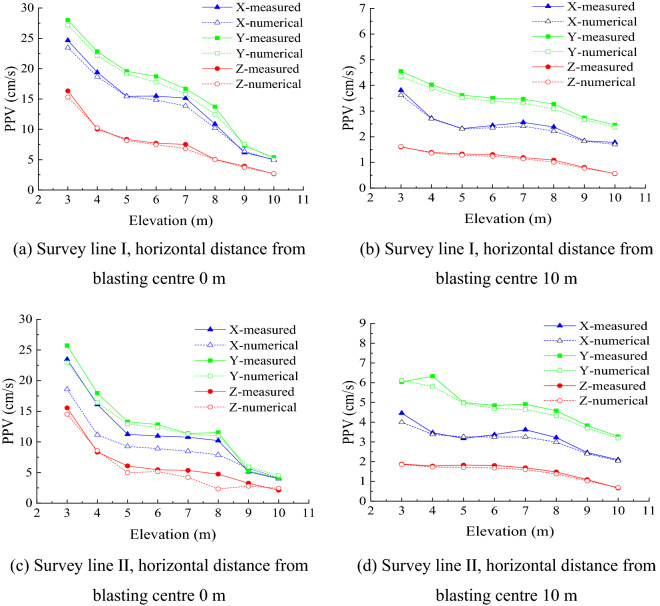
Comparison diagram of measured and numerically calculated PPV values.

The PPV at the survey points of the same positions as the on-site test was extracted and compared with the site survey points, as shown in Fig. 8. The measured PPV curve was consistent with the PPV curve from the numerical simulation, indicating that the numerical simulation results were reliable. The PPV in all directions had central amplification, and $V_{Y} > V_{X} > V_{Z}$.

**Figure 9:**
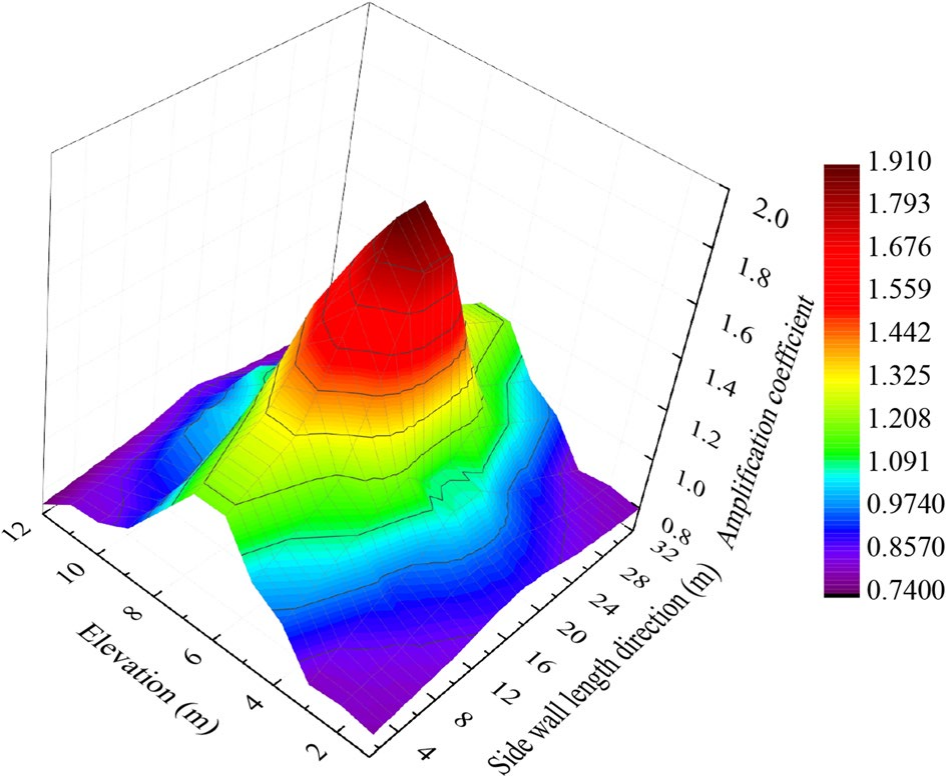
Amplification coefficient distribution diagram.

To study the distribution characteristics of the amplification effect on the sidewall, we extracted the PPV at all nodes on the sidewalls in the whole model cavern over time The PPV was extracted and divided by the corresponding value predicted by Sadovsky’s vibration formula to obtain the sidewall PPV amplification coefficient, as shown in Fig. 9. It can be seen from Fig. 9 that the PPV sidewall blasting amplification coefficient had a drum distribution. The amplification coefficient in the middle section on each survey line in the sidewall length direction was greater than 1, with a maximum of 1.91. The amplification coefficients close to the left and right ends of the sidewalls were much smaller than that in the middle section, indicating that the left and right ends of the sidewalls were subject to greater constraints. However, the amplification coefficients on the upper and lower ends in the elevation direction of sidewalls were smaller than 1, indicating that the underground sidewall blasting vibration waves were subject to greater constraints than those in semi-infinite planes. Therefore, the influence of constraints on the four sides and at the upper and lower ends on the sidewall PPV should be considered when conducting a theoretical study of the underground sidewall blasting vibration PPV distribution.

## Simply supported plate and beam mechanical analysis model and PPV prediction formula

Since the PPV prediction in the middle section of underground cavern high sidewalls by Sadovsky’s vibration formula are consistent with actual conditions, in this section we describe a mechanical analysis model more in line with actual conditions. The model considered structural dynamics in combination with high sidewall constraint conditions during underground blasting excavation. In addition, the vibration characteristics and dimensional methods were analysed to obtain a prediction formula suitable for blasting vibrations in the middle sections of high sidewalls.

### Analysis of the vibration response in the mechanical model of a simply supported plate

**Figure 10:**
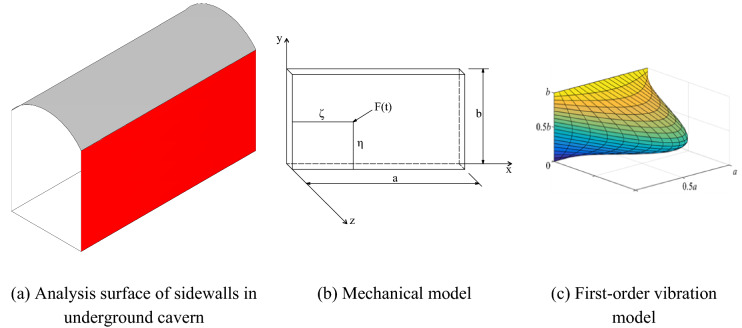
Simplified calculation model of the underground cavern sidewall.

Under actual engineering conditions, the upper and lower ends of the surface rock surrounding underground cavern sidewalls are subject to constraints from the roof and the bottom plate, while the left and right ends are subject to constraints from the boundaries. Therefore, the surrounding rock typically has simply supported plates on four sides, but are different from those of ordinary four-sided simply supported plates. Simplified conditions were used in this study to analyse such characteristics, as shown in Fig. 10a, the total length of the cavern was equivalent to that of plate a, and the total height was equivalent to that of plate b. The simplified mechanical model is shown in Fig. 10b, with the sidewall length direction as the X-axis, the sidewall height direction as the Y-axis, and the cavern width direction as the Z-axis. The first-order vibration mode is shown in Fig. 10c.

We simplified the four-sided constraints and the lateral forces on the surface surrounding rock. Since the explosion source was near the bottom of the sidewall, the blasting load was equivalent to a simple harmonic concentrated load F(x, y, t) acting on any point (x, y) on the simply supported plate on four sides. The forced vibration equation for the simply supported plate on four sides is:

2 $$\frac{{\partial^{4} w}}{{\partial x^{4} }} + 2\frac{{\partial^{4} w}}{{\partial x^{2} y^{2} }} + \frac{{\partial^{4} w}}{{\partial y^{4} }} - \frac{{\overline{m} }}{D}\frac{{\partial^{2} w}}{{\partial t^{2} }} = \frac{F(x,\;y,\;t)}{D}$$

where w is the displacement in the z-axis direction; $\overline{m}$ is the mass per unit area of the plate; D is the bending stiffness of the plate, $D = \frac{{Eh^{3} }}{{12(1 - \mu^{2} )}}$; and E and μ are the elasticity modulus and Poisson’s ratio of the material, respectively.

For the blast impact loads, when not considering the system damping effect, it can be assumed that:

3 $$\left. {\begin{array}{*{20}c} {F(x,\;y,\;t) = q(x,\;y)\sin \omega t} \\ {w(x,\;y,\;t) = w(x,\;y)\sin \omega t} \\ \end{array} } \right\}$$

where q(x, y) is the amplitude of the disturbance force; w(x, y) is the deflection surface amplitude equation; and ω is the driving frequency.

Then, the oscillatory differential equation changes to:

4 $$\frac{{\partial^{4} w}}{{\partial x^{4} }} + 2\frac{{\partial^{4} w}}{{\partial x^{2} y^{2} }} + \frac{{\partial^{4} w}}{{\partial y^{4} }} - \overline{m} \frac{{\omega^{2} }}{D}w = \frac{q(x,\;y)}{D}$$

When the dynamic basic solution of the deflection surface equation of the simply supported rectangular plate on four sides acts as a simple harmonic transverse unit concentrated load on any point (ζ, η) on the plate^46^:

5 $$w(x,\;y,\;\xi ,\;\eta ) = \frac{4}{Dab}\sum\limits_{m = 1}^{\infty } {\sum\limits_{n = 1}^{\infty } {\frac{1}{{K_{mn} }}} } \sin \frac{m\pi \eta }{a}\sin \frac{n\pi \xi }{b} \cdot \sin \frac{m\pi x}{a}\sin \frac{n\pi y}{b}\sin \omega t$$

where, $$K_{mn} = \left( {\frac{{m^{2} \pi^{2} }}{{a^{2} }} + \frac{{n^{2} \pi^{2} }}{{b^{2} }}} \right) - \overline{m} \frac{{\omega^{2} }}{D}$$.

It is not difficult to generalize from the dynamic basic solution that when the force F(t) at any point (ζ, η) on the simply supported rectangular plate on four sides is not a unit harmonic force, the deflection surface equation is:

6 $$w(x,\;y,\;\xi ,\;\eta ) = \frac{4}{Dab}\sum\limits_{m = 1}^{\infty } {\sum\limits_{n = 1}^{\infty } {\frac{1}{{K_{mn} }}} } \sin \frac{m\pi \eta }{a}\sin \frac{n\pi \xi }{b} \cdot \sin \frac{m\pi x}{a}\sin \frac{n\pi y}{b}C_{mn} (t)$$

where, $C_{mn} (t)$ is the amplitude function.

The partial differential of the time t was calculated by Eq. (5). The PPV of any point on the plate at any time was obtained:

7 $$\frac{\partial w(x,y,t)}{{\partial t}} = \frac{4}{Dab}\sum\limits_{m = 1}^{\infty } {\sum\limits_{n = 1}^{\infty } {\frac{1}{{K_{mn} }}} } \sin \frac{m\pi \eta }{a}\sin \frac{n\pi \xi }{b} \cdot \sin \frac{m\pi x}{a}\sin \frac{n\pi y}{b}C_{mn}^{^{\prime}} (t)$$

Generally, for rock and soil structures with higher stiffness, such as underground caverns, only the first-order vibration mode is considered. Moreover, for each blast, the load acting on point (ζ, η) is a known point; namely, for each blast, $\frac{\omega }{{K_{11} }}\sin \frac{\pi \eta }{a}\sin \frac{\pi \zeta }{b}$ is a known quantity. Thus, the ratio of the PPV at any point on the plate to the maximum velocity peak on the whole plate can be obtained: $\sin (m\pi x/a)\sin (n\pi y/b)$ and can be used as a dimensionless quantity to characterize the ratio of the PPV at any survey point on the sidewall to the maximum velocity peak on the whole sidewall.

### Analysis of the vibration response in the mechanical model of a simply supported beam

**Figure 11:**
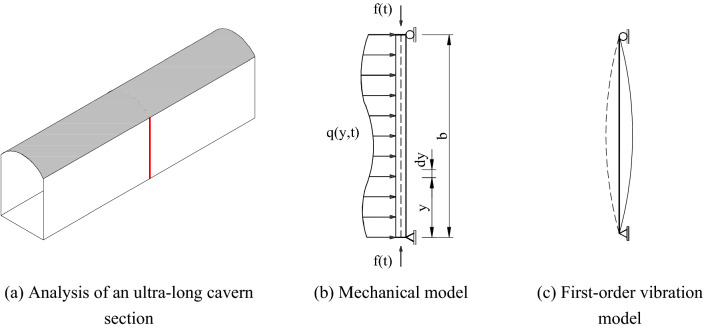
Simplified calculation of the ultra-long cavern model.

We analysed the section of the underground cavern sidewalls with a greater length–height ratio, as shown in Fig. 11a. Since a certain section of the wall was constrained by the upper and lower ends, it could be simplified to a simply supported beam for analysis. The applied load could be equivalent to the axial load and the transverse load. The latter is provided by the inner surrounding rock. During the explosion, any position on the beam is different at different times; thus, the transverse load can be equivalent to a function $q(y,t) = q(y)\sin (p_{1} t)$ of position and time. The axial load is jointly provided by the dynamite explosion and the surrounding rock at both ends and changes with time in the form of $f(t) = A\sin (p_{2} t)$, where $p_{1}$ and $p_{2}$ are driving frequencies. The total length of the cavern was equivalent to the total length of beam b. The simplified model is shown in Fig. 11b. To date, the vibration of cavern sidewalls has been simplified to the dynamic response of simply supported beams under biaxial loads. The first-order vibration mode of the simply supported beam is shown in Fig. 11c.

When only the deflection of the beam caused by the bending moment is considered and the thickness of the selected surrounding rock is small, the plane cross-section assumption of the Euler–Bernoulli beam is satisfied. $f(t)$ is a constant value at any time *t*. The differential equation for the bending vibration of a simply supported beam can be expressed by the D’Alembert principle:

8 $$EI\frac{{\partial^{4} Y}}{{\partial y^{4} }} + \overline{m} \frac{{\partial^{2} Y}}{{\partial t^{2} }} + f(t)\frac{{\partial^{2} Y}}{{\partial y^{2} }} = q(y)\sin (p_{1} t)$$

where $EI$ is the bending stiffness of the beam, $\overline{m}$ is the unit length mass of the beam, and $Y$ is the displacement function $Y(y,\;t)$.

The initial conditions of beam vibration are:

9 $$\left. {\begin{array}{*{20}c} {Y(y,t)\left| {_{t = 0} } \right. = 0} \\ {Y^{\prime}(y,t)\left| {_{t = 0} } \right. = 0} \\ \end{array} } \right\}$$

The boundary conditions of beam vibration are:

10 $$\left. {\begin{array}{*{20}c} {Y(0,t) = Y(b,t) = 0} \\ {Y^{\prime\prime}(0,t) = Y^{\prime\prime}(b,t) = 0} \\ \end{array} } \right\}$$

The displacement equation solution form of Eq. (7) is obtained by separating variables:

11 $$Y(y,t) = \sum\limits_{i = 1}^{n} {C_{n} } (t)\sin \frac{n\pi y}{b}$$

The influence of the high-order vibration mode is ignored. Only the first-order vibration mode is considered.

When $$n = 1$$,

12 $$Y(y,t) = C_{1} (t)\sin \frac{\pi y}{b}$$

The equation above is used to obtain the partial differential of time $t$ and the vibration velocity at any position at any time.

13 $$\frac{\partial Y(y,t)}{{\partial t}} = C_{1} ^{\prime}(t)\sin \frac{\pi y}{b}$$

From the above equation, we can obtain the maximum vibration velocity of the simply supported beam appears in the middle of beam $(1/2)H$ at any time with a value of $C_{1} ^{\prime}(t)$. The ratio of the vibration velocity $C_{1} ^{\prime}(t)\sin (\pi y/b)$ at any point on the beam to the maximum velocity peak $C_{1} ^{\prime}(t)$ on the whole beam is $\sin (\pi y/b)$. This ratio is a dimensionless quantity and can be used to characterize the influence of boundary constraints on the vibration velocity in a constrained space.

### Dimensional analysis of sidewall PPV considering four-side and two-end constraints

Combined with the dynamic analysis results in “Analysis of the vibration response in the mechanical model of a simply supported plate”, we see that the PPV of any survey point on the surface of the underground cavern blasting sidewall was related to the X-coordinate of the survey point $x$, the total length of the cavern $a$, the Y-coordinate of the survey point $y$, the total height of the sidewall $b$, and the sine function of $x/a$ and $y/b$. In addition, according to the traditional blasting vibration response analysis, blasting vibration was also mainly influenced by relevant factors such as topography and geomorphology, geological conditions, the maximum single shot dose $Q$, the distance from the survey point to the blasting centre $R$, the rock mass natural vibration frequency $f$, vibration wave propagation velocity $c$ and detonation time $t$, surface rock mass particle vibration displacement $\mu$, surface rock mass particle vibration acceleration $\overline{a}$, and rock mass density $\rho$. According to dimensional analysis, the underground cavern blasting sidewall PPV can be expressed as:

14 $$V = \Phi (Q,\;R,\;a,\;x,\;b,\;y,\;f,\;c,\;t,\;\mu ,\;\overline{a} ,\;\rho )$$

According to the number of parameters, there is a total of 12 physical quantities analysed. The independent variable is (Q, R, c) according to the π theorem, and there are 9 π components. $\pi_{i}$ represents a dimensionless quantity, then:

15 $$\left. {\begin{array}{*{20}c} {\pi_{0} = \frac{V}{c},\;\pi_{1} = \frac{\mu }{R},\;\pi_{2} = \frac{{\overline{a} R}}{{c^{2} }},\;\pi_{3} = \frac{fR}{c},\;\pi_{4} = \frac{b}{R},} \\ {\pi_{5} = \frac{y}{R},\;\pi_{6} = \frac{a}{R},\;\pi_{7} = \frac{x}{R},\;\pi_{8} = \frac{{\rho R^{3} }}{Q},\;\pi_{9} = \frac{tc}{R}} \\ \end{array} } \right\}$$

Equation (14) is substituted into Eq. (13), then:

16 $$\frac{V}{c} = \phi \left( {\frac{\mu }{R},\;\frac{{\overline{a} R}}{{c^{2} }},\;\frac{fR}{c},\;\frac{b}{R},\;\frac{y}{R},\;\frac{a}{R},\;\frac{x}{R},\;\frac{{\rho R^{3} }}{Q},\;\frac{tc}{R}} \right)$$

In addition, the following dimensionless quantity can be obtained:

17 $$\left. {\begin{array}{*{20}c} {\pi_{10} = (\pi_{4} )^{ - 1} \pi_{5} = \left( \frac{R}{b} \right)\left( \frac{y}{R} \right) = \frac{y}{b}} \\ {\pi_{11} = (\pi_{6} )^{ - 1} \pi_{7} = \left( \frac{R}{a} \right)\left( \frac{x}{R} \right) = \frac{x}{a}} \\ \end{array} } \right\}$$

18 $$\pi_{12} = \sin \left( {\frac{\pi x}{a}} \right)\sin \left( {\frac{\pi y}{b}} \right)$$

19 $$\pi_{13} = \left( {\frac{{\sqrt[3]{\rho }}}{{\sqrt[3]{Q}/R}}} \right)\left[ {\sin \left( {\frac{\pi x}{a}} \right)\sin \left( {\frac{\pi y}{b}} \right)} \right]$$

Under the same site conditions, ρ and c are approximated as constants. Therefore, from Eq. (18) there is a functional relationship between V and $\left( {\frac{{\sqrt[3]{\rho }}}{{\sqrt[3]{Q}/R}}} \right)\left[ {\sin \left( {\frac{\pi x}{a}} \right)\sin \left( {\frac{\pi y}{b}} \right)} \right]$. Then, the function can be written as:

20 $$\ln V = \left[ {\alpha_{1} + \beta_{1} \ln \left( {\frac{{\sqrt[3]{Q}}}{R}} \right)} \right] + \left\{ {\alpha_{2} + \beta_{2} \ln \left[ {\sin \left( {\frac{\pi x}{a}} \right)\sin \left( {\frac{\pi y}{b}} \right)} \right]} \right\}$$

where $\alpha_{2} + \beta_{2} \ln \left[ {\sin \left( {\frac{\pi x}{a}} \right)\sin \left( {\frac{\pi y}{b}} \right)} \right]$ characterizes the influence of the four-side constraints of the sidewall on PPV. If this item is ignored, Sadovsky’s vibration formula can be solved as follows:

21 $$V = k\left( {\frac{{\sqrt[3]{Q}}}{R}} \right)^{{\beta_{1} }}$$

If the influence of the four-side constraints of the sidewall on PPV is considered, the following can be obtained by solving Eq. (13):

22 $$V = e^{{\alpha_{1} }} e^{{\alpha_{2} }} \left( {\frac{{\sqrt[3]{Q}}}{R}} \right)^{{\beta_{1} }} \left[ {\sin \left( {\frac{\pi x}{a}} \right)\sin \left( {\frac{\pi y}{b}} \right)} \right]^{{\beta_{2} }}$$

When $$k^{^{\prime}} = e^{{\alpha_{1} + \alpha_{2} }}$$, $$\beta_{1}^{^{\prime}} = \beta_{1}$$, $$\beta_{2}^{^{\prime}} = \beta_{2}$$, then:

23 $$V = k^{^{\prime}} \left( {\frac{{\sqrt[3]{Q}}}{R}} \right)^{{\beta_{1} }} \left[ {\sin \left( {\frac{\pi x}{a}} \right)\sin \left( {\frac{\pi y}{b}} \right)} \right]^{{\beta_{2} }}$$

where $\alpha_{1}$, $\alpha_{2}$, $k$ and $k^{^{\prime}}$ are coefficients considering geological factors, constraints on the four sides of the sidewalls, and topographic influence; $\beta_{1}$ and $\beta_{1}^{^{\prime}}$ are the blasting vibration PPV attenuation coefficients relevant to geological conditions; and $\beta_{2}$ and $\beta_{2}^{^{\prime}}$ are impact factors of constraints on the four sides of the sidewalls.

Similarly, considering the influence of the constraints at the upper and lower ends of the cavern sidewall on the PPV, the vibration velocity formula of the sidewall can be derived as follows:

24 $$V = k^{\prime\prime}\left( {\frac{{\sqrt[3]{Q}}}{R}} \right)^{{\beta_{1}^{^{\prime}} }} \left( {\sin \frac{\pi y}{b}} \right)^{{\beta_{2}^{^{\prime}} }}$$

where $k^{\prime\prime}$ is the coefficient considering geological factors, constraints at the upper and lower ends, and topographic influence; $\beta_{1}^{^{\prime}}$ is the blasting vibration PPV attenuation coefficient relevant to geological conditions; and $\beta_{2}^{^{\prime}}$ is the impact factor of constraints at the ends.

## Verification of accuracy and applicability of prediction formula

### Verification of accuracy of prediction formula

Unitary regression and binary regression analyses were performed on Sadovsky’s vibration formula and prediction Eqs. (23) and (24) according to the data on Survey Lines I and II in the Taohuazui mine. We obtained formula coefficients: $k = {128}{\text{.2}}$, $\beta = 1.{74}$, $k^{^{\prime}} = 53.06$, $\beta_{1} = 0.75$, $\beta_{2} = 1.11$, $k^{^{\prime\prime}} = {60}{\text{.32}}$, $\beta_{1}^{^{\prime}} = 0.{92}$ and $\beta_{2}^{^{\prime}} = 1.12$; $k = 86.34$, $\beta = 1.57$, $k^{^{\prime}} = 43.57$, $\beta_{1} = 0.39$, $\beta_{2} = 1.5$, $k^{^{\prime\prime}} = 43.34$, $\beta_{1}^{^{\prime}} = 0.88$ and $\beta_{2}^{^{\prime}} = 1.13$. The predicted PPV values at each survey point could be further obtained by substituting such parameters as single shot dose, the distance from blasting centre, and elevation difference corresponding to the data at each survey point into the prediction formula.

**Figure 12:**
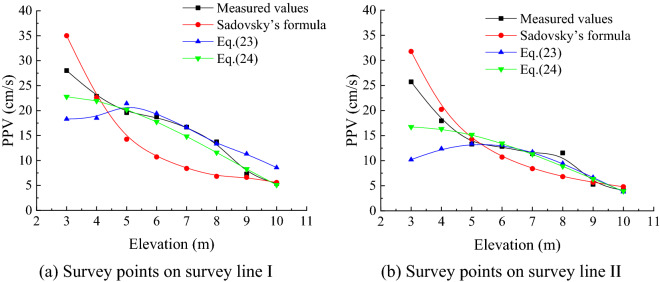
Comparative analysis of PPV at different elevations.

The variation in the measured value in the Y-axis direction at survey points on Survey Lines I and II, the value predicted by Sadovsky’s vibration formula, and the values predicted by the simply supported plate formula and simply supported beam formula with the elevation difference were drawn into curves on the same diagram for comparison (Fig. 12).

According to Fig. 12, the curve predicted by the simply supported plate formula was consistent within the elevation range of 5–8 m. The curve predicted by the simply supported beam formula also presents a certain degree of fitting within this range. However, the curve predicted by Sadovsky’s vibration formula had poor fitting. The fitting of Sadovsky’s vibration formula prediction curve within the elevation ranges of 3–4 m and 9–10 m with the measured curve was significantly higher than that of the curves predicted by the simply supported plate formula and the simply supported beam formula. This resulted from simplifying the boundary constraints in the mechanical analysis model when deriving the formula in this paper.

Since the dimensions of the selected underground cavern in the Taohuazui mine were limited to a length–height ratio less than 3, it was consistent with the mechanical model of the simply supported plate and not as consistent with the mechanical model of the simply supported beam. When the length–height ratio of the underground cavern sidewalls is great, the left and right constraint effects on the sidewall are insignificant. When the whole sidewall is simplified to the simply supported plate model, it is no longer applicable. Instead, the sidewall section should be simplified to a simply supported beam for analysis. The distribution characteristics of the PPV and amplification effect under different length–height ratios will be further studied.

### Applicability of the prediction formula considering the length–height ratio of an underground cavern

**Figure 13:**
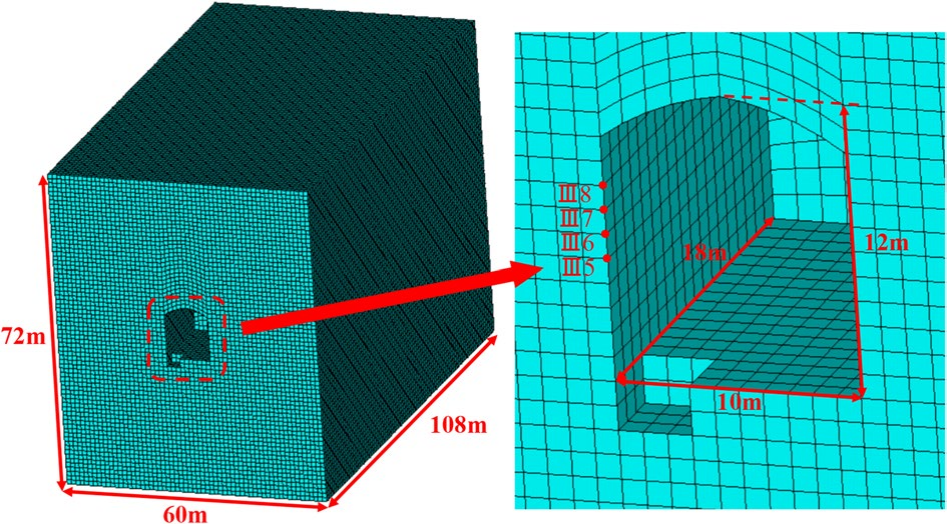
Section of the No. 2 numerical calculation model and details of the blasting test area.

We established a group of similar numerical models according to the parameters of the underground cavern in “Numerical simulation of the on-site test” to study the influence of the sidewall length–height ratio on PPV. Table 3 lists the parameters of the different numerical calculation models, and Fig. 13 gives the 1/2 section of the No. 2 numerical calculation model.

**Table 3:** Numerical calculation model parameters.

S. no.	_Height of cavern sidewall_ $b$ (m)	Length of cavern sidewall $a$ (m)	Length–height ratio	Ground stress *σ *(MPa)	Model length	Model width	Model height
1	12	12	1	20	144	60	72
2	12	36	3	20	216	60	72
3	12	60	5	20	360	60	72
4	12	84	7	20	504	60	72

**Figure 14:**
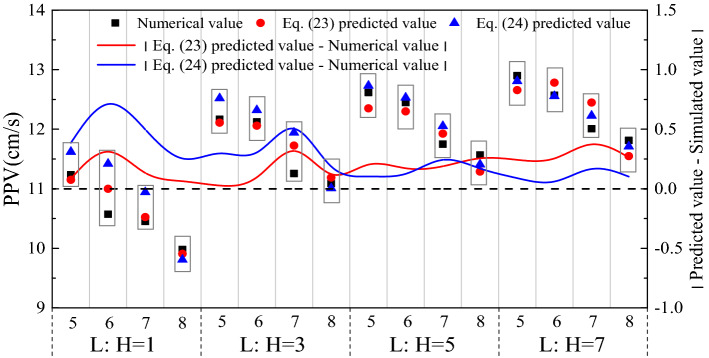
Influence of different length–height ratios on sidewall PPV and values predicted by two models.

The blasting zone of the four models was designed in the middle of the sidewall. The blasting PPV was selected as the survey line directly above the blast zones which were located at 6 m, 18 m, 30 m, and 42 m. A schematic of the blast zones and survey points is shown in Fig. 13. After completing calculations with the four models, the PPV data within the elevation difference range of 5–8 m on the survey line were extracted (Fig. 14). The fitting curves of Eqs. (23) and (24) were also drawn in the same diagram.

We see from Fig. 14 that with the increase in the sidewall length–height ratio, the PPV numerical simulation at the same elevation showed a slight increase. Since the blasting dose, blasting position, and distance from the blast centre were the same, the constraints of the left and right boundaries on the sidewall surrounding rock decreased with increasing sidewall length–height ratio. The decrease was manifested as an increase in sidewall PPV.

By comparing the closeness of the two absolute value curves of the difference between the predicted values and simulated values to the curve y = 0 we observed that the closer to y = 0, the better the prediction effect of the formula. In addition, when the length–height ratio of the sidewall was 1, 3 and 5, the closeness of the red curve to the curve y = 0 was basically the same and better than the blue curve. When the length–height ratio of the sidewall was 7, the red curve started to deviate from the curve y = 0. However, the blue curve became increasingly closer to the curve y = 0 with the increase in the length–height ratio and was very close to the red curve when the length–height ratio was 5. The blue curve was even closer to the curve y = 0 than the red curve when the length–height ratio was 7. This indicated that when the length–height ratio of the sidewall was greater than 7, the values predicted by the two-end constraint model were more accurate than those predicted by the four-side constraint model. Therefore, to ensure more accurate prediction results, we suggest using the mechanical model of a simply supported plate or simply supported beam and its prediction formula in the underground space with a sidewall length–height ratio of 5.

## Conclusion

In this paper, the four-side constraint and two-end constraint PPV prediction formulas were derived using an underground cavern blasting test in the Taohuazui mine in China with the mechanical model of a simply supported plate and simply supported beam. The following conclusions were drawn by comparing the measured sidewall PPV of the underground cavern with the PPV predicted by the formula:

1. The PPV on the underground cavern sidewall showed a “platform” or “bulge” at the sidewall middle elevation. That is, an elevation amplification effect appeared in the middle 1/3 section of the sidewall. The maximum amplification coefficient in the middle of the sidewall reached 1.9.
2. We found by comparing the prediction ability of Sadovsky’s vibration formula and the PPV prediction formula herein that the prediction formula considering “boundary constraints” could more accurately predict the PPV drum distribution characteristics in an underground cavern sidewall middle section.
3. When the length–height ratio of an underground cavern sidewall is smaller than 5, the mechanical model of the simply supported plate and its prediction formula are recommended.

The boundary conditions of an underground cavern sidewall surrounding rock are complicated. Therefore, we simplified the boundary constraints when establishing a mechanical analysis model, leading to some limitations in the use of the model and prediction formula. However, the end constraints on the surrounding rock are actually a kind of force constraint between simple and clamped supports. Further study on this problem should be done. Since it is difficult to obtain complete and accurate data on the blasting PPV of underground cavern high sidewalls, the mechanical models and prediction formulas proposed in this paper need to be tested in more projects.
